# Insights into the IgG heavy chain engineering patent landscape as applied to IgG4 antibody development

**DOI:** 10.1080/19420862.2019.1664365

**Published:** 2019-09-26

**Authors:** Christophe Dumet, Jérémy Pottier, Valérie Gouilleux-Gruart, Hervé Watier

**Affiliations:** a EA7501, Team ”Fc Receptors, Antibodies and Microenvironnement”, Université de Tours, France; b CHRU de Tours, France

**Keywords:** IgG4, Fc, engineering, patents

## Abstract

Despite being the least abundant immunoglobulin G in human plasma, IgG4 are used therapeutically when weak effector functions are needed. The increase in engineered IgG4-based antibodies on the market led us to study the patent landscape of IgG4 Fc engineering, *i.e*., patents claiming modifications in the heavy chain. Thirty-seven relevant patent families were identified, comprising hundreds of IgG4 Fc variants focusing on removal of residual effector functions (since IgG4s bind to FcγRI and weakly to other FcγRs), half-life enhancement and IgG4 stability. Given the number of expired or soon to expire major patents in those 3 areas, companies developing blocking antibodies now have, or will in the near future, access to free tools to design silenced, half-life extended and stable IgG4 antibodies.

## Introduction

Although the least abundant of the 4 IgG subclasses naturally found in human plasma,^1^ IgG4 antibodies comprise the second largest subclass of approved monoclonal antibody (mAb) products behind IgG1. Among the 80 approved biopharmaceuticals containing a human IgG Fc portion (70 mAbs and 10 fusion proteins), 58 are indeed based on IgG1, and 15 are based on IgG4, historically relying on a desire to avoid target cell killing and immune activation,^2^ while maintaining a plasmatic half-life as long as that of IgG1.

At one time, IgG4 were thought to be unable to bind to cellular Fc receptors and to induce antibody-dependent cell-mediated cytotoxicity (ADCC), and it was considered the worst subclass at promoting complement-induced target cell lysis.^3-8^ A direct comparison of IgG4 and IgG1 versions of Campath (anti-CD52 alemtuzumab) in a clinical study,^9^ however, showed that Campath-G4 depleted target cells in most patients, but to a lesser extent than Campath-G1.^9,10^ IgG4 were initially thought to bind to FcγRI only,^11^ but study results indicate they also bind to FcγRIIA, FcγRIIB, FcγRIIC, and FcγRIIIA, especially the V158 allotype, although less than IgG1.^12,13^ Indeed, the use of IgG4 does not guarantee the absence of immune activation, since cross-linking by FcγRIIB may have been critical for the “cytokine storm”^14^ observed in volunteers during the tragic TGN1412 (anti-CD28 superagonist IgG4) Phase 1 trial.

**Figure 1.**
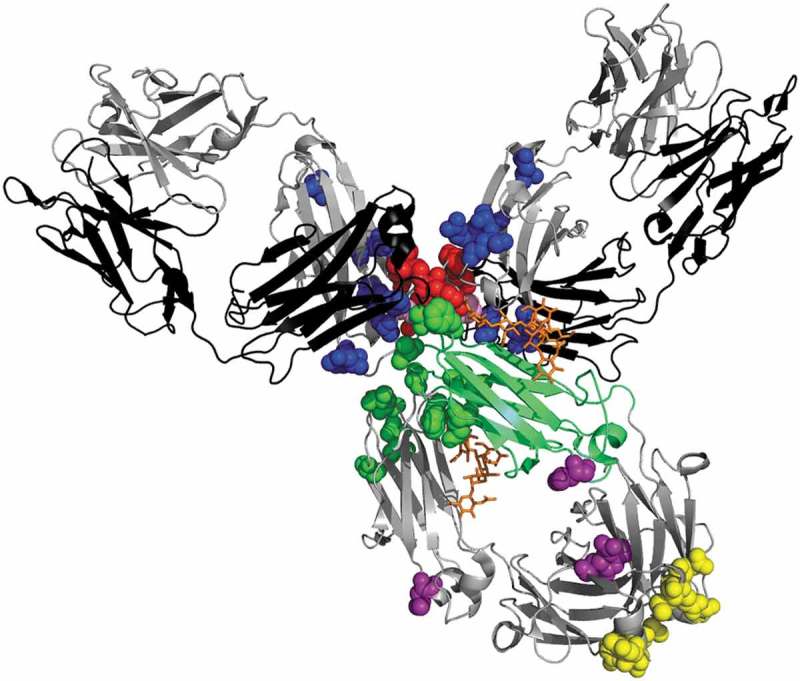
Primary structural differences between γ4 and γ1 heavy chains. (a) Three dimensional structure of pembrolizumab highlighting the amino-acid differences between γ4 and γ1 heavy chains: pembrolizumab differs from wild-type IgG4 by one amino-acid at position 228 in the hinge region, a proline replacing a serine residue (S228P, shown in pink but masked in part by other residues). The rotated CH2 of pembrolizumab is shown in green cartoon. The two glycans are indicated in orange, showing the external exposition of the glycan from the rotated domain. IgG4 presents two isoallotypes (single nucleotide polymorphisms in the *IGHG4* gene),^15^ at position 309 in CH2 with either a leucine or a valine, and at position 409 in the CH3 with either an arginine or a lysine, shown in purple spheres. The positions which have different amino-acid between γ4 and γ1 heavy chains are shown in blue sphere for CH1 (131; 133; 137; 138; 196; 199; 203; 214), in red spheres for hinge region (217; 219; 220; 224; 225; 228), in green spheres for CH2 (234; 268; 274; 296; 327; 330; 331), and in yellow spheres for CH3 (355; 356; 358; 409; 419; 445). All of these residues were superimposed on the pembrolizumab structure (PDB: 5DK3^16^) using PyMOL Molecular Graphics System, version 1.7.4 (Schrödinger). (b) Amino-acid sequence comparison between γ1 and γ4 hinge regions. Nucleotide and amino-acid differences in γ4 compared to γ1 are shown in red. Cysteines indicating disulfide bridges are shown in bold. The nucleotide alignment has enabled us to number amino acids at positions Y219 and G220, which were not numbered in the original Eu numbering. γ4 hinge region differ by 6 amino-acids from γ1 hinge region. The γ4 hinge has a three amino-acid deletions (shown in gray), with only two disulfide bridges, while γ1 has three. The missing one, C220 bridges the light chain to the γ1 heavy chain; in IgG4, this inter-chain bridge involves C131 in the CH1.^17,18.^

**Figure 2.**
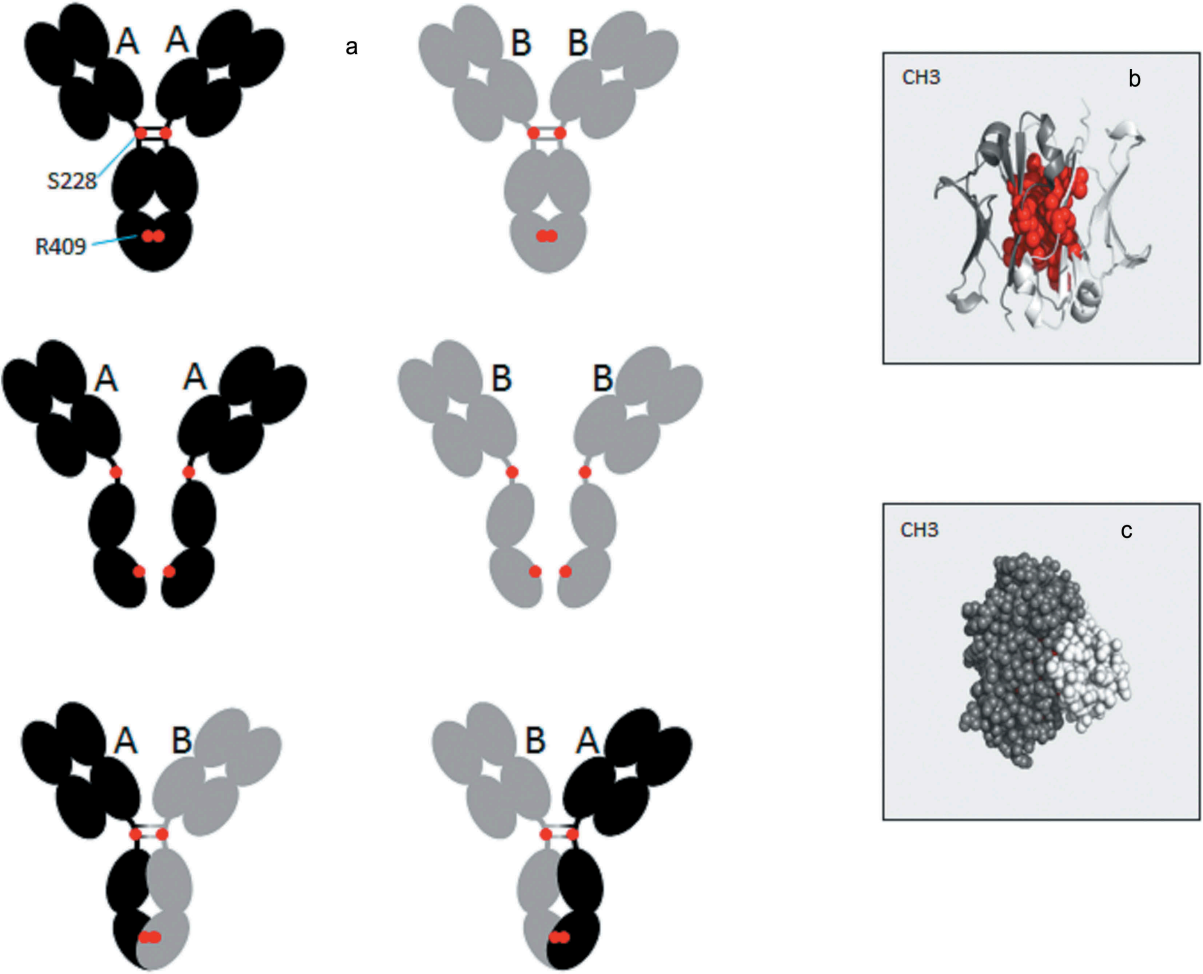
Schematic representation of the Fab-arm exchange phenomenon. (a) Under mild reducing and native conditions, wild type IgG4 show a dissociation process to form half-molecule both *in vitro* and *in vivo*. This half-molecule can reassemble with a heavy chain from another antibody, to form a bispecific antibody. Hotspots of modification reported in cartoon (b) or spheres (c) in the IgG4 CH3 structure (PDB: 4B53) using PyMOL Molecular Graphics System, version 1.7.4 (Schrödinger). The most frequently modified amino-acids (L351, T366, L368, K370, D399, F405, Y407, and R409) are shown in red spheres.

Pharmacological differences between IgG1 and IgG4 subclasses result from structural differences between the γ1 and γ4 heavy chains. However, depending on allotypes (genetic variants), comparison between these two isotypes can be misleading. Here, we use γ1 and γ4 to refer to the chains encoded by the IGHG1*01 and IGHG4*01 alleles, respectively. The differences are mainly located in and around the hinge (Figure 1(a,b)) and can also affect the downstream processing during bioproduction. For example, the purification process can contribute to aggregation, but to a different extent according to the subclass. Indeed, IgG4 have been showed to be less stable than IgG1s at low pH conditions.^19,20^ In particular, the serine at position 228 is specific to γ4 and is one of the key amino-acids playing a role in the stability of IgG4s. A study has shown that S228P substitution resulted in a homogenous IgG4 when analyzed by SDS-PAGE.^21^ It was later demonstrated that this substitution could block the half-IgG exchange phenomenon usually known as Fab-arm exchange (Figure 2).^22,23^

**Figure 3.**
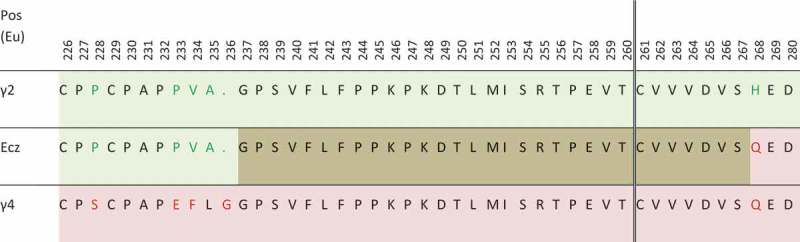
Partial sequence of eculizumab (Ecz), compared with sequences of γ2 (light green) and γ4 (light pink) heavy chains. Even if the fusion occurs after T260 in the patent (vertical double line), the overlapping area between γ2 and γ4 sequence is shown in brown. Amino-acids in green and red in γ2 and γ4 sequences are those differing between these subclasses.

Other γ1/γ4 differences are located in the heart of the CH3 and at its C-terminal side (Figure 1(a,b)). It was shown that not only the hinge region but also the CH3 domain contribute to Fab-arm exchange,^23^ and particularly the arginine residues at position 409,^24^ which is polymorphic since replaced by a lysine in the IGHG4*03 allele. The role of IgG4 Fab-arm exchange has been interpreted as a type of posttranslational modification, which could serve as an additional mechanism for generating anti-inflammatory activity.^23^ This exchange prevents IgG4s from bridging two membrane antigens or forming immune complexes with soluble antigens since exchanged IgG4 become bispecific, and thus functionally monovalent. This phenomenon does not seem to cause safety issues, based on the results for natalizumab (anti-α4 integrin), reslizumab (anti-IL-5) or ibalizumab (anti-CD4), which are wild-type approved IgG4 mAbs known to undergo Fab-arm exchange with endogenous IgG4.^22^

Of the 15 approved IgG4-based therapeutics (Table 1), 12 are modified in their constant heavy chain, while only 14 of 50 approved IgG1-based therapeutics harbor constant heavy chain modifications. Moreover, most of the IgG4 antibodies currently in Phase 3 bear constant heavy chain modifications (Table 1). The increase of engineered IgG4 constant heavy chain-based products arriving on the market, notably the anti-PD1 antibodies, led us to study the US and European patent landscape regarding technologies optimizing classical antagonist IgG4 antibodies. We discuss areas where companies are free to operate (or not), the relevance of different technologies, and what could be the standard IgG4 blocking antibody constant region format in the future.

**Table 1.** Biopharmaceuticals with an IgG4-Fc fragment that are approved or in late-stage clinical development.

Antibody	Target	Society	Format	Fc modifications (Eu)	Approval year or phase reached in the US
Gemtuzumab ozogamicin	CD33	Wyeth/Pfizer Celltech/UCB	IgG4	S228P	2000, withdrawn in 2010, approved in 2017
Natalizumab	VLA-4	Biogen	IgG4	No mutation	2006
Eculizumab	C5	Alexion	IgG2/IgG4	IgG2 until T260, then IgG4 (Figure 3)	2007
Dulaglutide	GLP-1 R	Lilly	IgG4 fusion protein	S228P, F234A, L235A^a^	2014
Nivolumab	PD1	BMS	IgG4	S228P	2015
Pembrolizumab	PD1	Merck	IgG4	S228P	2015
Ixekizumab	IL-17	Lilly	IgG4	S228P^a^	2016
Reslizumab	IL-5	Teva Celltech/UCB	IgG4	No mutation	2016
Emicizumab	Factor IX x Factor X	Chugai Roche	IgG4	S228P, K196Q F296Y E356K, R409K, H435R, L445P, G446> del, K447> del	2017
Inotuzumab ozogamicin	CD22	Pfizer/Celltech/UCB	IgG4	S228P	2017
Dupilumab	IL-4Rα	Regeneron/Sanofi	IgG4	S228P	2017
Ibalizumab	CD4	TaiMed Tanox Genentech (Roche)	IgG4	No mutation	2018
Cemiplimab	PD-1	Regeneron/Sanofi	IgG4	S228P	2018
Galcanezumab	CGRP	Lilly	IgG4	S228P, F234A, L235A^a^	2018
Ravulizumab	C5	Alexion	IgG2/IgG4	IgG2 until T260, then IgG4, M428L, N434S, K447> del	2018
Andecaliximab	MMP-9	Gilead	IgG4	S228P	III
Tralokinumab	IL-13	MedImmune	IgG4	No mutation	III
Camrelizumab	PD-1	Jiangsu HengRui Medicine/Incyte	IgG4	S228P	III
Spartalizumab	PD-1	Novartis	IgG4	S228P, K447> del	III
Tislelizumab	PD-1	BeiGene/Celgene	IgG4	S228P, E233P, F234V, L235A, D265A, R409K	III
Mirikizumab	IL-23	Lilly	IgG4	S228P, F234A, L235A, K447> del	III
Evinacumab	ANGPTL3	Regeneron/Bayer	IgG4	S228P	III
CS1001	PD-L1	Cstone Pharmaceuticals	IgG4	No sequence found	III
Lebrikizumab	IL-13	Roche/ Genentech	IgG4	S228P	III
Fasinumab	NGF	Regeneron/Sanofi	IgG4	S228P	III
Crenezumab	Aβ42 and Aβ40	Roche Genentech	IgG4	S228P	III
Leronlimab	CCR5	CytoDyn	IgG4	No mutation	II/III
Dostarlimab	PD-1	AnaptysBio/Tesaro, Inc.	IgG4	S228P	III
Narsoplimab	MASP-2	Omeros Corporation	IgG4	S228P	III
Rozanolixizumab	FcRn	UCB	IgG4	S228P	III
Olokizumab	IL-6	R-Pharm	IgG4	S228P	III
Sutimlimab	C1S	R-Pharm	IgG4	S228P,L235E	III
Relatlimab	LAG-3	Medarex	IgG4	S228P,K447> del	II/III

Marketed antibodies are in gray cases; ^a^These variants also include a deletion of the lysine at position 447 (and emicizumab also a deletion at position 446), abrogating the heterogeneity at the C-terminal end; ^b^In the anti-factor IX chain: K196Q, S228P, F296Y, E356K, R409K, H435R, L445P and removal of G446 and K447^a^, in the anti-factor X chain: A196Q, S228P, F296Y, R409K, K439E, L445P and removal of G446 and K447^a^

## Patent analysis

### Description of the patent corpus

A corpus of patent families integrating the structural and functional description of IgG4 constant heavy chain modifications was established by using relevant keywords, cooperative patent classification codes or citing patents in the Orbit© patent database (Questel, France). The claims sections were carefully examined to build a patent corpus referring to the main engineering improvements of antagonist IgG4 antibodies. The areas of improvements were: 1) reduction of effector functions, 2) half-life modulation, 3) stability, and 4) downstream processes. Because issues like half-life modulation or effector function reductions are not only related to IgG4, the corpus includes broader patents, covering any polypeptide comprising an IgG framework. Here, we define broad claims as main claims covering any polypeptide comprising at least an IgG4-Fc with the disclosed technology. Main claims covering any antibody directed to a particular target or having particular variables domains in association with an engineering technology were referred as restrictive. Many applications describe variants that were not, or are no longer, protected by a patent. Close examination of the details of what has been described and what has been claimed is generally necessary because the patent landscape is always evolving. New family patents need to be monitored, but also applications or new applications in current patent families. Indeed, continuation applications may be issued later and what has not been claimed in an earlier application could be claimed in a later application of the same family in order to obtain the full protection extent of the parent application. For example, Xencor filed several continuations in part from a 2003 parent application (WO2004029207) and the last application from this family was issued in 2018.

As of June 2019, the corpus comprised 40 patent families incorporating claims encompassing IgG4 framework modifications. Patents in the same restricted family (determined by Orbit) were grouped according to the first international publication number (if existing) of the family (Table 2).

**Table 2.** Applications related to IgG4 heavy chain modifications addressed in the text^a.^

Family Application Number^a^	Main claim^b^	Main Applicant	Filing year	US patent (if any)	expiration date	EU patent (if any)	expiration date
**Effector Functions Reduction**							
WO8907142	Replacement of hinge, CH2, or CH3 with the ones from other subclasses	UNIV COLUMBIA/BECTON DICKINSON	1989			REVOKED	
WO9428027	Replacement of L235 and F234 to abrogate effector functions	MACROGENICS/JANSSEN	1993	EXPIRED	2019	EXPIRED	
WO9429351	Antibodies with reduced effector functions, including an IgG4 with L235E	CELLTECH	1993			LAPSED	
WO9526403	Stabilized (S228P) antibody against E-selectin with weak effector functions (L235A)	CELLTECH	1994	LAPSED		EXPIRED	
WO200042072	Multiple mutations (main claims are sometimes very broad since neither amino acid substituted nor functions effect are specified	GENENTECH	2000	GRANTED	2020	GRANTED	2020
WO2004029207/US20060024298	At least positions 328, A330R, T299A	XENCOR	2003	GRANTED	2024		
WO2005018572	Aglycosylated antibody (T299A,C)	BIOGEN	2004	GRANTED	2024	GRANTED	2024
WO2005007809	An antibody composed of a portion of IgG2 (until T260) fused to IgG4 to abrogate effector functions	ALEXION	2004	LAPSED		LAPSED	
WO2011066501	S228P/F234A/L235A/G237A/P238S	CENTOCHOR/JANSSEN	2011	GRANTED	2035		
WO2011149999	F243A/V264A	MERCK and Co	2011	GRANTED	2032		
WO2012130831	S228P/L235E/P329G	ROCHE	2012	GRANTED	2032	GRANTED	2032
WO2014121087	Multiple mutation and domain swapping	REGENERON	2014	GRANTED	2034	GRANTED	2034
WO2017079369	Multiple mutations	GSK	2016				
**Half-Life Enhancement**							
WO200042072	N434, T307 substitution	GENENTECH	2000	GRANTED	2020	GRANTED	2020
WO02060919	M252Y/S254T/T256E mutations and several other mutation combination	MEDIMMUNE	2001	GRANTED	2022	GRANTED	2021
WO2004035752	T250Q/E and M428L/F combination	ABBOT	2003	GRANTED	2024	GRANTED	2023
WO2006053301	N434S, V308W, V308Y, V308F	XENCOR	2005	GRANTED	2026	GRANTED	2026
WO2007114319	Isoelectric point modification	CHUGAI	2007			REVOKED	
WO2009058492	M252Y/M428L, D259I/V308F, N434S	XENCOR	2007	GRANTED	2030	GRANTED	2028
WO2009086320	M428L/N434S	XENCOR	2008			GRANTED	2028
WO2010045193	Several mutations	GENENTECH	2009	PENDING		PENDING	
WO2010106180	Multiple mutation combination	LFB	2009	GRANTED	2030	GRANTED	2030
US20100204454	M428L/N434S,T307Q/N434S, M428L/V308F, and Q311V/N434S	XENCOR	2010	GRANTED	2028		
WO2012016227	Mutation combination lowering isoelectric point	XENCOR	2011	GRANTED	2031		
WO2013074598	Mutations already known in combination with mutations in position F243/V264	MERCK and Co	2013				
GB201302878	H433K/N434F	ARGENX	2013			LAPSED	
WO2013163630	E258F/V427T	BIOATLA	2013				
US20140294812	Multiple mutation combination	XENCOR	2014				
WO2015175874	Modifications at positions 432 and 437	MEDIMMUNE	2015				
WO2017158426	Mutation combinations at positions 311, 428, 434, 435 and 438.	University of Oslo	2017				
WO2018052556	No mutation claimed (only list of properties), multiple combinations mutations in dependent claims like T256D/Q311V/A378V	Visterra	2017				
KR101792191, KR101792205, KR20180113717, KR20180113907, KR20180113904	Multiple mutation combinations	University of Kookmin	2017				
US20190010243	K288E/H435K	Macrogenics	2018				
**Downstream Processing**							
WO2006033386	S228P/L235E/R409K,T,M,L	KYOWA HAKKO KIRIN	2005	GRANTED	2025	GRANTED	2025
WO2008145142	R409K,T,M,L and hinge without CPPC motif	GENMAB	2008			GRANTED	2027
WO2009041613	G446/del/K447del	CHUGAI	2009	GRANTED	2029	GRANTED	2029
WO2010085682	Multiple mutations reducing the aglycosylation-induced loss of thermal stability	BIOGEN	2010	LAPSED		LAPSED	
WO2010063785	IgG4 stabilized with mutations K370Q/E and optionally R409X/L309X	GENMAB	2010	GRANTED	2029	GRANTED	2029
WO2012022982	C131 and upper modifications	UCB	2010			GRANTED	2031
US2017029521	K370 substitution	GENMAB	2016				
WO2018065389	Mutations in the region between Kabat residues 203 and 256 reducing binding to HCP	GSK	2017				
WO2018119380	Substitution at positions 197 or 217/220/(224 or 225)	BMS	2017				

^a^The table is organized around parent applications (generally PCT).

^b^ Main claims in the table are the most relevant claims regarding an IgG4 blocking antibody. Note that the precise content of claims and the number of granted patents may differ between the US and Europe.

### Patents related to IgG effector function reduction

One of the first applications regarding modulation of IgG properties (WO8907142) was filed in 1989 by Columbia University. The idea was to swap the domains of different IgG subclasses in order to obtain antibodies with desired properties. Although a European patent was granted in 1996, Celltech filed an opposition and the patent was eventually revoked in 2006.

The first application (WO9428027) claiming a silenced IgG4 (with the L235E substitution) was filed in 1993 by Arch Development Corporation. However, the US and European patents main claims are quite restrictive since they refer to an anti-CD3 antibody with this mutation, and not to any antibody having this mutation. The example section of other applications (WO9429351, WO9526403) contain IgG4 variants with mutations (L235A, F234A or G237A). However, we did not find any patent with a broad claim covering any IgG (hence IgG4) comprising a single mutation at either of these positions to abolish effector functions. Celltech filed an application (WO9526403) in 1994 with a broad claim regarding reduction of complement activation, but no patent was granted. Indeed, several publications^10,25^ had already identified the binding sites associated with effector functions. In 2000, Genentech filed an application (WO200042072) comprising hundreds of single variants that later resulted in dozens of patents. For the most part, the patents relate to FcyR binding enhancement, but they also contain examples of mutations reducing the binding to all FcyR, e.g., D265A, which is covered by a patent (US7332581) in the family; however, its legal status is uncertain. These patents are due to expire in 2020. In 2003, Xencor filed several applications from their parent application (WO2004029207) comprising hundreds of Fc variants modulating affinity to FcγR. Among them, they obtained US patents claiming, for example, the L328 substitution decreasing ADCC, or the A330R mutation decreasing binding to FcγRIIIA. Some have been opposed, including the EP2364996B1 patent covering the F243L mutation.

Another way to abolish effector functions is to mutate residues in or close to the *N*-glycosylation site,^26,27^ as claimed in WO2005018572. However, although references to aglycosylated IgG4 may be found in patents, we did not find any aglycosylated IgG4 mAb in development.

From the early 1990s to the early 2000s, publications and patent applications disclosed most of the interesting single mutations reducing effector functions. Companies are thus seeking to patent mutation combinations in order to freely exploit their own silenced mAbs.

Alexion developed a technology to abolish effector functions by joining an IgG2 (up to T260) with the end of an IgG4 Fc (Figure 3), giving a molecule with very weak binding to C1q and Fcγ receptors. This is the format of the anti-C5 marketed antibodies eculizumab (Soliris®) and ravulizumab (ULTOMIRIS®). Alexion withdrew their 2005 application (WO2005007809) describing this format, probably due to their prior publication in 1997, which compromised any claim of novelty.^28^ However, to our knowledge, this format (albeit not covered by a patent) has not yet been used by other companies, at least for mAbs in clinical development (except eculizumab biosimilars). However, patent applications comprising this kind of IgG2/IgG4 format^29^ have been issued, including the WO2015140591 application by the Norwegian University of Science and Technology and the WO2011066501 application by Centocor. In the latter, it was originally an IgG2-based format with IgG4 point mutations,^30^ but Janssen (formerly Centocor) eventually obtained a patent (US10053513B2) concerning a mutated IgG4 (S228P, F234A, L235A, G237A, and P238S) from the same WO2011066501 patent family.

Filed in 2011, Merck and Co.’s application WO2011149999 described sialylated Fc-polypeptides comprising the F243A/V264A mutation combination. Although this mutation combination is related to an IgG1 framework in the example section and in the claims of the granted patent, these mutations could be useful, if they result in a more reduced binding to Fcγ receptors, with an IgG4 framework.

P329G substitution combined with L234A and L235A, in the case of IgG1, or S228P and L235E, in the case of IgG4, has been showed to further decrease effector functions^31^ by disrupting a sandwich proline motif within the Fc/Fcγ receptor interface. Surprisingly, IgG1 antibodies bearing these mutations were more silenced than IgG4. Roche started to exploit this technology through an IgG1 framework (cergutuzumab amunaleukin (anti-CEA immunocytokine)). In 2015, Roche was granted a US patent from the family application WO2012130831 for any polypeptide comprising an Fc, including this mutations combination. In 2017, Xencor also claimed the P329G substitution, but they apparently abandoned their application (US2017166655). In 2014, Regeneron applied for claims related to antibodies with reduced effector functions and was granted both US and European patents from the WO2014121087 family. Although the main claim covers any antibody, the protection is narrow since it covers several heavy chain modifications, which are not independent from each other. Surprisingly, the main claim in the European patent specifies that the antibody can bind with higher affinity (although weakened by the mutations) to FcγRIIA than to FcγRIIB. GlaxoSmithKline’s 2016 application WO2017079369 described new IgG2 Fc and IgG4 Fc mutation combinations, especially the E233P/F234A/L235A/G236del/G237A mutation combination for IgG4.

### Patents related to IgG4 half-life modulation

Half-life is critical for any IgG subclass used as a therapeutic, since its extension could help decrease the amount dosed or the spacing of administrations, for example. Therefore, having a patent portfolio regarding technologies applicable to any kind of polypeptide containing an Fc portion is of the utmost importance. Given the large number of family patents issued in the last decades, a non-exhaustive list of documents will be discussed hereafter.

Genentech was among the first applicants to obtain patents regarding mutations that enhance the binding of human antibodies to FcRn. From the WO200042072 patent family, they obtained three patents, covering the 307, 380 and 434 positions. For example, N434A, N434H or T307A/E380A/N434A improved half-life in animal models,^32,33^ with N434A being probably the best variant. However, the patent claiming a mutation at position 434 only covers IgG1 and is deemed to expire in 2020.

Another pioneer, Medimmune (now AstraZeneca) has probably the most interesting patent portfolio. In 2001, from the WO02060919 application family, MedImmune obtained a US patent (US7083784) with broad main claims covering modifications at eight positions 252/254/256/309/311/433/434/436. This patent covers the well-known M252Y/S254T/T256E (YTE)^34^ mutation combination, which increases the serum half-life of antibodies in cynomolgus monkeys by nearly 4-fold^34^ and increases the half-life of motavizumab up to 100 days in humans.^35^ Seven other patents from this family were granted, extending protection to other mutation combinations located or near the Fc/FcRn interface (308/311/385/386/389/428). The European patents are different since the main claim in patent EP1355919B1 protects the single 252 position (Y,F,W,T modifications) and the main claim of the EP2354149B1 patent only protects the 433K/434F/436H combination. This patent family is deemed to expire in 2022.

From the WO2004035752 application family, Abbott was granted both a US and a European patent regarding any antibody comprising the T250Q/E and M428L/F substitutions.^36^ Antibodies of different subclasses and different CDRs including the mutation combination T250Q/M428L (QL) showed a near 2-fold IgG half-life enhancement in rhesus macaques.^37^

Xencor has many broad patents from several application families, with both US and European patents for: 1) the N434S, 308W, 308Y and 308F mutations (WO2006053301 family, expires in 2026), 2) the M252Y/M428L and D259I/V308F combination (WO2009058492), and 3) their well-known M428L/N434S (LS) (WO2009086320) mutation combination reported as having 4-fold extended half-life in humans^38^ (deemed to expire in 2028). Xencor’s patent portfolio also includes patents covering the T307Q/N434S, M428L/V308F, and Q311V/N434S combinations.

In 2009, LFB was granted a US patent (WO2010106180) regarding 12 mutation combinations enhancing FcRn binding. Surprisingly, several mutations are not located at the Fc/FcRn interface, but some of them, such as prolines at positions 228 and 230 could have distant effects on the FcRn binding site. However, given the differences between the hinge regions of IgG1 and IgG4, it is unclear whether the effect of these mutations would be the same for IgG1 variants. Monnet et al.^39^ evaluated the pharmacokinetics of 6 mutants in huFcRn transgenic mice and showed they had half-lifes up to 2.8-fold superior to the wild type, but we did not find any *in vivo* data for humans administered antibodies with these mutations.

In 2009, Genentech filed a patent application (WO2010045193) to protect mutation combinations for positions already described in earlier applications, notably combinations comprising the T307Q/N434A mutations, which results in a 25-day half-life in cynomolgus monkeys. Despite documents questioning novelty, US and European applications have not been abandoned yet.

Other organizations have tried to apply for different Fc mutations enhancing the binding to FcRn, such as Bioatla in 2013 (WO2013163630). They issued several applications with different mutations, for example, the E258F/V427T mutation combination in the (US20180186863) application. However, there is no description of their mutants in their application; the example section only states that the tested variants have a 2- to 80-fold FcRn binding at acidic pH compared to the wild type Fc and normal binding at neutral pH. Visterra, University of Oslo (WO2017158426), University of Kookmin (KR101792191 multiple applications) and Macrogenics (US20190010243) also found new variants with enhanced properties. For example, Visterra^40^ (WO2018052556) designed new variants with enhanced half-life while retaining good effector functions (although this feature is not relevant for a blocking antibody).

Serum half-life could also be decreased by lowering the isoelectric point^41^ via substitution of positively charged amino acids for ones with negative charges. For example, Chugai was granted a broad European patent (EP2006381) regarding serum half-life modulation through any modification in the variable region that alters the isoelectric point. Alexion, followed by others, opposed the patent and Chugai’s patent was eventually revoked, but Chugai appealed the decision. From the WO2012016227 parent application, Xencor also has one EU (EP3029066) and two US (US8697641 and US9605061) patents covering isoelectric point modification in the constant regions, but they are restricted to particular mutation combinations. Other applications, such as recently issued US20180222965, claim mutation combinations modulating half-life, but it is still too soon to know if these claims will be maintained.

### Patents related to stabilization and downstream processing

Celltech researchers had published in 1992^21^ that a single S228P substitution (proline being present at this position in all IgG subclasses but IgG4) in the hinge region was sufficient to abolish heterogeneity of human IgG4 antibodies. In 1994, Celltech filed a patent application (WO9526403) for an antibody against E-selectin, comprising the L235E substitution that decreases effector functions and the S228P substitution abolishing the formation of half antibody molecules. However, to our knowledge, this well-known mutation has never been protected as such. Indeed, we did not find any patent containing a broad claim regarding the S228P substitution that would encompass any IgG4 mAbs and yet, this discovery has had a major impact on the development of IgG4-derived antibodies. This mutation is nearly always included in newly developed IgG4 antibodies. For example, the companies that developed tabalumab (anti-BAFF) or relatlimab (anti-LAG-3) had planned to incorporate this mutation since their patents claimed or mentioned S228P as an embodiment of the invention. Although this mutation solved the main problem of IgG4 stability, companies tried to patent alternative solutions.

IgG4 stabilization patents moved from focusing on the hinge to the CH3 in 2006, with a Kyowa application (WO2006033386) addressing the issue of IgG4 aggregation under low pH conditions by mutating R409. In the examples section, they demonstrated that S228P/L235E/R409K variants showed less of a tendency to aggregate at low pH than S228P/L235E variants. Both US and European patents were granted with broad claims regarding mutation combinations for inhibiting IgG4 aggregation. Genmab’s application (WO2008145142) in 2008 described an identical mutation reducing Fab-Arm exchange,^42^ but an opposition was filed against their European patent, mainly due to the prior Kyowa patent. Genmab’s patent was maintained, although amended, because of its reference to the hinge region in the claim, and because the opposition division made a distinction between the Fab-arm exchange phenomena and the aggregation process. The opposition division acknowledged that the novel and inventive step was Genmab’s demonstration that the 409 mutation alone (hence not necessarily with the S228P) could reduce Fab-arm exchange. Made one year later, Genmab’s application (WO2010063785) for other stabilizing substitutions such as K370Q/E (among others) was granted both US and European patents. In 2016, they also filed an application (US2017029521) for any substitution at position 370, but limited to polypeptides comprising a CXPC or CPXC (not S228P) sequence in the hinge region. We did not find any patent covering the S228P/R409K mutation combination, which could combine both advantageous effects. As a matter of fact, companies had already considered using this format for further developments. For instance, in the example section of application WO2018127586A1, Elsalys Biotech tested this mutation combination for their anti-CD160 mAb. Other companies like Calypso considered this format in the embodiment of their EP2985295A1 application, concerning their anti-MMP9 mAb.

In 2010, Biogen filed a patent application (WO2010085682) for a technology involving aglycosylated, stabilized IgG4 with IgG1 CH3 domains. The idea was to engineer a silent IgG with numerous mutations reducing the aglycosylation-induced loss of thermal stability. They described numerous mutations, for example at positions 297, 299, 307, 309, 399, 409 and 427, as well as valine substitutions (in 240, 262, 264, and 266) to hydrophobic amino acids associated with greater bulk in a hydrophobic patch. However, they withdrew the application.

The S228P is not the only mutation that can both modulate disulfide bond formation and stabilization of the molecule.^43^ In 2010, UCB filed an application (WO2012022982) and was granted a European patent for any IgG4 with a substitution at position 131 associated with a substitution of any amino acid to cysteine in the upper hinge. They generated numerous IgG4 variants with increased Fab thermal stability and reduced product heterogeneity by modifying the upper hinge in addition with the S228P mutation. However, it is not clear whether these new mutations give a real advantage compared to the sole S228P mutation.

Chugai was granted a European patent from the WO2009041613 family, which covers the reduction of IgG4 C-terminal heterogeneity^44^ by deleting G446 and K447. According to their sequence listing and their claims, the protection applies to any IgG4 having the S228P mutation. Although Chugai has a patent on C-terminal heterogeneity reduction by deleting both G446 and K447 amino acids, deletion of only K447 is already used in some antibodies, such as blosozumab (anti-sclerostin), dupilumab (Dupixent®, anti-IL-4Rα), emibetuzumab (anti-cMet), ixekizumab (Taltz®, anti-IL-17). This mutation taken alone does not seem to be covered by any patent. However, according to Chugai patent WO2009041613, its double deletion could further diminish heterogeneity compared to the sole K447deletion.

According to application WO2018119380 filed by Bristol-Myers Squibb in 2017, S228P-stabilized IgG4 can cause undesirable bio-analytical and bioprocessing behaviors. In the example section, they showed that S228P IgG4 tends to elute as a double peak on CEX-HPLC, probably caused by two binding conformations in the column. To limit this behavior, mutations have been introduced into the heavy chain. The main claim is the substitution of lysine at position 196 by any other amino acid or substitution at positions 217/220/(224 or 225).

In addition, Tran et *al*. showed that IgG4 were more prone than IgG1 to bind to individual host cell proteins such as phospholipase B-like 2.^45^ In 2017, GlaxoSmithKline filed an application (WO2018065389) covering mutations in the heavy chain (more specifically to the hinge region) that abolish the binding to this protein, and more broadly to host cell proteins. This application is in an early stage, and it’s unlikely the very broad main claim will be granted, since numerous documents have questioned novelty.

## Developments and perspectives

Although IgG4 have weak effector functions, their binding to FcγRs show that they are not totally devoid of immune activities. Patent applicants have focused on this issue since the early 1990s, and, for example, dozens of patent families describe variants in the CH2 with modified effector functions. However, few applications include *in vitro* comparison with mutations already patented, making it difficult to assess the pros and cons of a particular mutation. For example, few data are available about the effects of a given mutation on the yield and quality of the antibody production. In the context of IgG4 development, freedom to operate regarding reducing effector functions is not of great concern, as there are several technologies not covered by any patent. Only particular mutations or mutation combinations are protected by broad claims (like Roche’s patent from WO2012130831) and the advantage over well-established technologies that are freely available for use remains to be demonstrated.

In contrast, the mutations that increase half-life in animal models the most (YTE, QL and LS) are still covered by patents. At least 3 antibodies containing the YTE mutations, suvratoxumab (MEDI4893, anti-*Staphylococcus aureus* alpha toxin), MEDI8897 (anti-RSV) and MEDI5117 (anti-IL-6), are or were in development, and at least 5 antibodies containing the LS (Xtend technology) mutation are in development or approved, including Alexion’s ravulizumab (Ultomiris®, anti-C5), according to Xencor’s pipeline. Because the patents of Medimmune (now AstraZeneca) and Xencor are broad, it is difficult to assess which mutations can be freely used and which cannot. However, the YTE mutation is deemed to fall in the public domain in 2022, and QL and LS in 2024 and 2028, respectively. Currently, an antibody with the YTE mutation combination seem to have the most notably improved pharmacokinetic properties, as validated in a human trial,^35^ although new variants also have good pH6.0/pH7.0 ratio binding.^46^ Moreover, YTE mutations seem to reduce effector functions, which could be of interest for pure antagonistic IgG mAbs. As pointed out by Borok et al.,^46^ further enhancement in half-life may be difficult, and other ways, in addition to FcRn binding modulation, may be required to improve the half-life of an antibody.

In addition to incorporating technologies already freely available, companies could start the development of antibodies comprising mutations deemed to fall in the public domain in the near future. Under the research privilege (“Bolar exemption”), studies and trials that provide data needed for marketing approval do not infringe patents. For example, efgartigimod (human Fc targeting FcRn) comprises YTE in its combination of mutations, but there is no patent infringement as long as the product reaches the market after expiry of patents covering one of those mutations (for example, the EP1355919B1 patent covering the M252Y/W/F/T).

**Figure 4.**
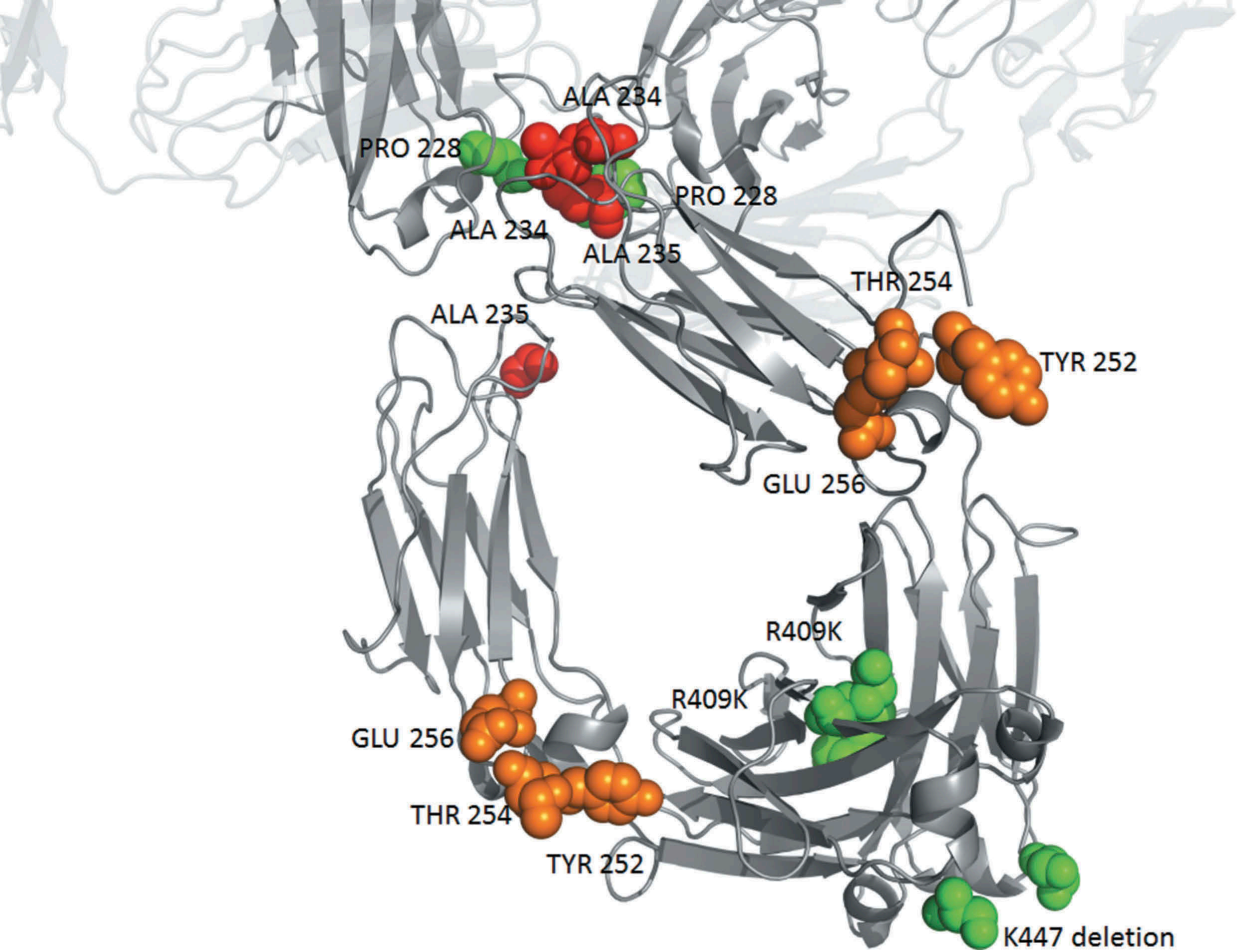
Possible heavy chain mutations combination for an optimized IgG4 blocking antibody being soon free to operate. Mutations modulating effector functions, half-life and stability are shown in red, orange and green respectively. For illustrating 447 deletion, the last amino-acid (serine 444) of this particular structure is represented in green. Pembrolizumab structure (PDB: 5DK3^16^) using PyMOL Molecular Graphics System, version 1.7.4 (Schrödinger).

Given the patent landscape and scientific purpose, companies can choose to incorporate modifications or not in the constant region of an antibody. In the context of a blocking IgG4 antibody, it is tempting to design the “perfect” antibody optimized in 3 aspects: reduced effector function, increased half–life, and improved downstream processing. Hence, future IgG4 blocking antibodies may well comprise YTE, silencing mutations, R409K and K447del (Figure 4). This is already the case, in part, for several antibodies containing the K447del and/or F234A/L235A mutations in addition to S228P, such as mirikizumab (anti-Il-23, Phase 3), galcanezumab (Emgality®, anti-CGRP, approved) and emibetuzumab (anti-cMet, Phase 2), which were developed by Eli Lilly. It is possible that too many mutations could impair stability, yield or cause immunogenicity. However, several highly mutated IgG4 antibodies (e.g., tislelizumab, emicizumab, ravulizumab) have advanced to Phase 3 or were approved, as have many other antibodies that are of different subclasses. Our data suggest that the number of mutated IgG4 will increase, as will the number of mutations per antibody.

## References

[1] Morell A, Skvaril F, Steinberg AG, Van Loghem E, Terry WD. Correlations between the concentrations of the four sub-classes of IgG and Gm Allotypes in normal human sera. J Immunol [Internet] 1972;108:195–206. http://www.ncbi.nlm.nih.gov/pubmed/4622006. 4622006

[2] Salfeld JG. Isotype selection in antibody engineering. Nat Biotechnol [Internet] 2007 [cited 2016 118];25:1369–72. doi:10.1038/nbt1207-1369. 18066027

[3] Ishizaka T, Ishizaka K, Salmon S, Fudenberg H Biologic activities of aggregated gamma-globulin. 8. Aggregated immunoglobulins of different classes. J Immunol. 1967;99:82–91. 4166026

[4] Brüggemann M, Williams GT, Bindon CI, Clark MR, Walker MR, Jefferis R, Waldmann H, Neuberger MS Comparison of the effector functions of human immunoglobulins using a matched set of chimeric antibodies. J Exp Med. 1987;166:1351–61. doi:10.1084/jem.166.5.1351. 3500259 PMC2189658

[5] Bindon CI, Hale G, Brüggemann M, Waldmann H Human monoclonal IgG isotypes differ in complement activating function at the level of C4 as well as C1q. J Exp Med [Internet] 1988;168:127–42. doi:10.1084/jem.168.1.127. 3260935 PMC2188986

[6] Riechmann L, Clark M, Waldmann H, Winter G Reshaping human antibodies for therapy. Nature. 1988;332:323–27. doi:10.1038/332323a0. 3127726

[7] Tao MH, Smith RI, Morrison SL Structural features of human immunoglobulin G that determine isotype-specific differences in complement activation. J Exp Med. 1993;178:661–67. doi:10.1084/jem.178.2.661. 8340761 PMC2191116

[8] Schumaker VN, Calcott MA, Spiegelberg HL, Mueller-Eberhard HJ Ultracentrifuge studies of the binding of IgG of different subclasses to the Clq subunit of the first component of complement. Biochemistry. 1976;15:5175–81. doi:10.1021/bi00668a035. 990273

[9] Isaacs JD, Wing MG, Greenwood JD, Hazleman BL, Hale G, Waldmann H A therapeutic human IgG4 monoclonal antibody that depletes target cells in humans. Clin Exp Immunol. 1996;106:427–33. doi:10.1046/j.1365-2249.1996.d01-876.x. 8973608 PMC2200623

[10] Greenwood J, Clark M, Waldmann H. Structural motifs involved in human IgG antibody effector functions. Eur J Immunol [Internet] 1993;23:1098–104. 10.1002/eji.1830230518 8477804

[11] Ravetch JV, Kinet JP Fc receptors. Annu Rev Immunol. 1991;9:457–92. doi:10.1146/annurev.iy.09.040191.002243. 1910686

[12] Koene HR, Kleijer M, Algra J, Roos D, von Dem Borne AE, de Haas M Fc gammaRIIIa-158V/F polymorphism influences the binding of IgG by natural killer cell Fc gammaRIIIa, independently of the Fc gammaRIIIa-48L/R/H phenotype. Blood. 1997;90:1109–14. 9242542

[13] Bruhns P, Iannascoli B, England P, Mancardi DA, Fernandez N, Jorieux S, Daëron M Specificity and affinity of human Fcgamma receptors and their polymorphic variants for human IgG subclasses. Blood. 2009;113:3716–25. doi:10.1182/blood-2008-03-146472. 19018092

[14] Hussain K, Hargreaves CE, Roghanian A, Oldham RJ, Claude Chan HT, Mockridge CI, Chowdhury F, Frend??us B, Harper KS, Strefford JC, et al Upregulation of F??RIIb on monocytes is necessary to promote the superagonist activity of TGN1412. Blood. 2015;125:102–10. doi:10.1182/blood-2014-07-591040. 25395427

[15] Brusco A, Saviozzi S, Cinque F, DeMarchi M, Boccazzi C, De Lange G, Van Leeuwen AM, Carbonara AO Molecular characterization of immunoglobulin G4 gene isoallotypes. Eur J Immunogenet [Internet] 1998;25:349–55. doi:10.1046/j.1365-2370.1998.00113.x. 9805657

[16] Scapin G, Yang X, Prosise WW, McCoy M, Reichert P, Johnston JM, Kashi RS, Strickland C Structure of full-length human anti-PD1 therapeutic IgG4 antibody pembrolizumab. Nat Struct Mol Biol [Internet] 2015 [cited 2017 31];22:953–58. doi:10.1038/nsmb.3129. 26595420

[17] Frangione B, Milstein C, Pink JRL Immunoglobulins: structural Studies of Immunoglobulin G. Nature. 1969;221:145–48. doi:10.1038/221145a0. 5782707

[18] Liu H, May K Structural variations, chemical modifications and possible impacts to stability and biological function Disulfide bond structures of IgG molecules © 2012 Landes Bioscience. MAbs. 2012;4:17–23. doi:10.4161/mabs.4.1.18347. 22327427 PMC3338938

[19] Neergaard MS, Nielsen AD, Parshad H, Van De Weert M Stability of monoclonal antibodies at high-concentration: head-to-head comparison of the IgG1 and IgG4 subclass. J Pharm Sci [Internet] 2014;103:115–27. doi:10.1002/jps.23788. 24282022

[20] Ejima D, Tsumoto K, Fukada H, Yumioka R, Nagase K, Arakawa T, Philo JS Effects of acid exposure on the conformation, stability, and aggregation of monoclonal antibodies. Proteins [Internet] 2007;66:954–62. doi:10.1002/prot.21243. 17154421

[21] Angal S, King DJ, Bodmer MW, Turner A, Lawson AD, Roberts G, Pedley B, Adair JR A single amino acid substitution abolishes the heterogeneity of chimeric mouse/human (IgG4) antibody. Mol Immunol. 1993;30:105–08. doi:10.1016/0161-5890(93)90432-B. 8417368

[22] Labrijn AF, Buijsse AO, van Den Bremer ETJ, Verwilligen AYW, Bleeker WK, Thorpe SJ, Killestein J, Polman CH, Aalberse RC, Schuurman J, et al Therapeutic IgG4 antibodies engage in Fab-arm exchange with endogenous human IgG4 in vivo. Nat Biotechnol. 2009;27:767–71. doi:10.1038/nbt.1553. 19620983

[23] van der Neut Kolfschoten M, Schuurman J, Losen M, Bleeker WK, Martínez-Martínez P, Vermeulen E, Den Bleker TH, Wiegman L, Vink T, Aarden LA, et al Anti-inflammatory activity of human IgG4 antibodies by dynamic Fab arm exchange. Science. 2007;317:1554–57. doi:10.1126/science.1144603. 17872445

[24] Davies AM, Rispens T, Den Bleker TH, McDonnell JM, Gould HJ, Aalberse RC, Sutton BJ Crystal structure of the human IgG4 CH3 dimer reveals the role of Arg409 in the mechanism of Fab-arm exchange. Mol Immunol. 2013;54:1–7. doi:10.1016/j.molimm.2012.12.019. 23164605

[25] Sarmay G, Lund J, Rozsnyay Z, Gergely J, Jefferis R Mapping and comparison of the interaction sites on the Fc region of IgG responsible for triggering antibody dependent cellular cytotoxicity (ADCC) through different types of human Fc gamma receptor. Mol Immunol. 1992;29:633–39. doi:10.1016/0161-5890(92)90200-H. 1533898

[26] Tao MH, Morrison SL Studies of aglycosylated chimeric mouse-human IgG. Role of carbohydrate in the structure and effector functions mediated by the human IgG constant region. J Immunol. 1989;143:2595–601. 2507634

[27] Walker MR, Lund J, Thompson KM, Jefferis R Aglycosylation of human IgG1 and IgG3 monoclonal antibodies can eliminate recognition by human cells expressing Fc gamma RI and/or Fc gamma RII receptors. Biochem J. 1989;259:347–53. doi:10.1042/bj2590347. 2524188 PMC1138517

[28] Mueller JP, Giannoni MA, Hartman SL, Elliott EA, Squinto SP, Matis LA, Evans MJ Humanized porcine VCAM-specific monoclonal antibodies with chimeric IgG2/G4 constant regions block human leukocyte binding to porcine endothelial cells. Mol Immunol. 1997;34:441–52. doi:10.1016/S0161-5890(97)00042-4. 9307060

[29] Lau C, Gunnarsen KS, Høydahl LS, Andersen JT, Berntzen G, Pharo A, Lindstad JK, Ludviksen JK, Brekke O-L, Barratt-Due A, et al Chimeric anti-CD14 IGG2/4 hybrid antibodies for therapeutic intervention in pig and human models of inflammation. J Immunol [Internet] 2013;191:4769–77. doi:10.4049/jimmunol.1301653. 24062486 PMC3804170

[30] An Z, Forrest G, Moore R, Cukan M, Haytko P, Huang L, Vitelli S, Zhao JZ, Lu P, Hua J, et al IgG2m4, an engineered antibody isotype with reduced Fc function. MAbs. 2009;1:572–79. doi:10.4161/mabs.1.6.10185. 20073128 PMC2791314

[31] Schlothauer T, Herter S, Koller CF, Grau-Richards S, Steinhart V, Spick C, Kubbies M, Klein C, Umaña P, Mössner E Novel human IgG1 and IgG4 Fc-engineered antibodies with completely abolished immune effector functions. Protein Eng Des Sel. 2016;29:457–66. doi:10.1093/protein/gzw039. 27578889

[32] Petkova SB, Akilesh S, Sproule TJ, Christianson GJ, Al Khabbaz H, Brown AC, Presta LG, Meng YG, Roopenian DC Enhanced half-life of genetically engineered human IgG1 antibodies in a humanized FcRn mouse model: potential application in humorally mediated autoimmune disease. International Immunology [Internet] 2006;18:1759–69. doi:10.1093/intimm/dxl110. 17077181

[33] Deng R, Loyet KM, Lien S, Iyer S, Deforge LE, Theil F-P, Lowman HB, Fielder PJ, Prabhu S Pharmacokinetics of humanized monoclonal anti-TNF{alpha} antibody and its FcRn variants in mice and cynomolgus monkeys. Drug Metab Dispos [Internet] 2010;38:600–05. doi:10.1124/dmd.109.031310. 20071453

[34] Dall’Acqua WF, Kiener PA, Wu H Properties of human IgG1s engineered for enhanced binding to the neonatal Fc receptor (FcRn). J Biol Chem [Internet] 2006;281:23514–24. doi:10.1074/jbc.M604292200. 16793771

[35] Robbie GJ, Criste R, Dall’Acqua WF, Jensen K, Patel NK, Losonsky GA, Griffin MP A novel investigational Fc-modified humanized monoclonal antibody, motavizumab-YTE, has an extended half-life in healthy adults. Antimicrob Agents Chemother. 2013;57:6147–53. doi:10.1128/AAC.01285-13. 24080653 PMC3837853

[36] Hinton PR, Johlfs MG, Xiong JM, Hanestad K, Ong KC, Bullock C, Keller S, Tang MT, Tso JY, Vásquez M, et al Engineered human IgG antibodies with longer serum half-lives in primates. J Biol Chem [Internet] 2004;279:6213–16. doi:10.1074/jbc.C300470200. 14699147

[37] Datta-Mannan A, Witcher DR, Lu J, Wroblewski VJ Influence of improved FcRn binding on the subcutaneous bioavailability of monoclonal antibodies in cynomolgus monkeys. MAbs [Internet] 2012;4:267–73. doi:10.4161/mabs.4.2.19364. 22377715 PMC3361662

[38] Gaudinski MR, Coates EE, Houser KV, Chen GL, Yamshchikov G, Saunders JG, Holman LSA, Gordon I, Plummer S, Hendel CS, et al Safety and pharmacokinetics of the Fc-modified HIV-1 human monoclonal antibody VRC01LS: A Phase 1 open-label clinical trial in healthy adults. PLoS Med. 2018;15:1–20. doi:10.1371/journal.pmed.1002593. PMC5783347 29364886

[39] Monnet C, Jorieux S, Souyris N, Zaki O, Jacquet A, Fournier N, Crozet F, De Romeuf C, Bouayadi K, Urbain R, et al Combined glyco- and protein-Fc engineering simultaneously enhance cytotoxicity and half-life of a therapeutic antibody. MAbs. 2014;6:422–36. doi:10.4161/mabs.27854. 24492301 PMC3984331

[40] Booth BJ, Ramakrishnan B, Narayan K, Wollacott AM, Babcock GJ, Shriver Z, Viswanathan K Extending human IgG half-life using structure-guided design. MAbs [Internet] 2018:1–13. doi:10.1080/19420862.2018.1490119. PMC6204840 29947573

[41] Igawa T, Tsunoda H, Tachibana T, Maeda A, Mimoto F, Moriyama C, Nanami M, Sekimori Y, Nabuchi Y, Aso Y, et al Reduced elimination of IgG antibodies by engineering the variable region. Protein Eng Des Sel. 2010;23:385–92. doi:10.1093/protein/gzq009. 20159773

[42] Labrijn AF, Rispens T, Meesters J, Rose RJ, Den Bleker TH, Loverix S, van Den Bremer ETJ, Neijssen J, Vink T, Lasters I, et al Species-specific determinants in the IgG CH3 domain enable fab-arm exchange by affecting the noncovalent CH3-CH3 interaction strength. J Immunol. 2011;187:3238–46. doi:10.4049/jimmunol.1100967. 21841137

[43] Peters SJ, Smales CM, Henry AJ, Stephens PE, West S, Humphreys DP Engineering an improved IgG4 molecule with reduced disulfide bond heterogeneity and increased fab domain thermal stability. J Biol Chem. 2012;287:24525–33. doi:10.1074/jbc.M112.369744. 22610095 PMC3397877

[44] Harris RJ Processing of C-terminal lysine and arginine residues of proteins isolated from mammalian cell culture. J Chromatogr A. 1995;705:129–34. doi:10.1016/0021-9673(94)01255-D. 7620566

[45] Tran B, Grosskopf V, Wang X, Yang J, Walker D, Yu C, McDonald P Investigating interactions between phospholipase B-Like 2 and antibodies during Protein A chromatography. J Chromatogr A [Internet] 2016;1438:31–38. doi:10.1016/j.chroma.2016.01.047. 26896920

[46] Borrok MJ, Wu Y, Beyaz N, Yu XQ, Oganesyan V, Dall’Acqua WF, Tsui P PH-dependent binding engineering reveals an FcRn affinity threshold that governs IgG recycling. J Biol Chem. 2015;290:4282–90. doi:10.1074/jbc.M114.603712. 25538249 PMC4326836

